# Depth and coral cover drive the distribution of a coral macroborer across two reef systems

**DOI:** 10.1371/journal.pone.0199462

**Published:** 2018-06-20

**Authors:** Rebecca L. Maher, Michelle A. Johnston, Marilyn E. Brandt, Tyler B. Smith, Adrienne M. S. Correa

**Affiliations:** 1 Rice University, BioSciences at Rice, Houston, Texas, United States of America; 2 Oregon State University, Department of Microbiology, Corvallis, Oregon, United States of America; 3 National Oceanic and Atmospheric Administration–Flower Garden Banks National Marine Sanctuary, Galveston, Texas, United States of America; 4 Center for Marine and Environmental Studies, University of the Virgin Islands, 2 John Brewers Bay, Saint Thomas, Virgin Islands, United States of America; Pennsylvania State University, UNITED STATES

## Abstract

Bioerosion, the removal of calcium carbonate from coral frameworks by living organisms, influences a variety of reef features, from their topographic complexity to the net balance of carbonate budgets. Little is known, however, about how macroborers, which bore into reef substrates leaving traces greater than 0.1 mm diameter, are distributed across coral reefs, particularly reef systems with high (>50%) stony coral cover or at mesophotic depths (≥30 m). Here, we present an accurate and efficient method for quantifying macroborer densities from stony coral hosts via image analysis, using the bioeroding barnacle, *Lithotrya dorsalis*, and its host coral, *Orbicella franksi*, as a case study. We found that in 2014, *L*. *dorsalis* densities varied consistently with depth and host percent cover in two Atlantic reef systems: the Flower Garden Banks (FGB, northwest Gulf of Mexico) and the U.S. Virgin Islands (USVI). Although average barnacle density was nearly 4.5 times greater overall in the FGB than in the USVI, barnacle density decreased with depth in both reef regions. Barnacle density also scaled negatively with increasing coral cover in the study areas, suggesting that barnacle populations are not strictly space-limited in their distribution and settlement opportunities. Our findings suggest that depth and host coral cover, and potentially, local factors may strongly influence the abundance of macroborers, and thus the rate of CaCO_3_ loss, in a given reef system. Our image analysis method for quantifying macroborers can be standardized across historical and modern reef records to better understand how borers impact host growth and reef health.

## Introduction

If mechanical and biological erosive processes equal or exceed reef carbonate production, reef framework may be destroyed faster than it is produced [1], resulting in a net negative carbonate budget [2]. On many modern reefs, calcium carbonate accretion barely exceeds destruction [2–4], particularly in systems that frequently experience anthropogenic disturbance. Evidence of this can be seen in the Caribbean, where current carbonate production rates are 50% lower than long-term rates and increasing numbers of reefs are considered net erosional [4]. Shifts toward net erosion are of great concern because they can jeopardize the biodiversity, ecosystem functions, and environmental services associated with reefs [5].

Biological erosion (bioerosion) is a biologically mediated process that occurs when micro- and macro-organisms physically break or chemically dissolve reef framework [5]. Macroborers, including taxa such as worms, sponges, barnacles, and bivalves have boring diameters greater than 0.1 mm [6,7]. These organisms can significantly reduce the net calcium carbonate budget on reefs [3,8] and the longevity of individual coral colonies by weakening their carbonate skeletons [9,10]. However, widespread reductions in bioerosion rates due to population declines of some borers have partially offset net carbonate production declines [11]. Understanding the impact of bioerosion is critical for predicting future carbonate budget dynamics and supporting the persistence of healthy, structurally complex reef systems [12].

Although macroborers have received some research attention due to their role in the calcium carbonate budget [3,5,8] and their potential use as bioindicators [1,13], there is little to no consensus regarding how the distribution, density, and activity of macroborers varies with depth [1,14–19]. Quantitative bioerosion data for modern reefs rarely encompass shallow and mesophotic depth ranges and may present conflicting patterns [18]. For example, evidence exists for both a decrease [3,16,20–23] and an increase in borer activity with depth [14,17]. Studies investigating bioerosion by the polychaete *Spirobranchus giganteus* with host coral colony area have also reported conflicting results. Hunte *et al*. [21] found no effect of colony size on polychaete density, whereas Floros *et al*. [24] and Dai and Yang [25] found that density scales positively with colony surface area. Differences could be related to coral host identity as *S*. *giganteus* is distributed non-randomly among coral species [25]. Additional quantitative data are needed to better resolve how different boring taxa are distributed with depth and available coral substrate. From this baseline, other patterns can be examined, such as how borers respond to natural and anthropogenic disturbance.

Although there is strong consensus that bioeroding taxa such as excavating sponges and echinoids can significantly reduce net carbonate production on a reef [5,26,27], other borers are assumed to remove negligible amounts of reef calcium carbonate. For example, various studies characterizing coral reef bioeroding communities have reported that burrowing barnacles are rare or absent in corals and that they play a small role in the erosion of coral reefs [1,28–30], yet other studies report barnacle bioeroding contributions greater than 10% of total bioerosion (Table 1). Given this, and the lack of published studies quantifying barnacle distributions on mesophotic reefs (Table 1), we investigated the density of the barnacle macroborer, *Lithotrya dorsalis*, on the dominant reef-building coral, *Orbicella franksi*, in both shallow (<30 m) and mesophotic (≥30 m) depths in two regions: the Flower Garden Banks National Marine Sanctuary (FGBNMS, northwest Gulf of Mexico) and the U.S. Virgin Islands (USVI, Caribbean). USVI reefs have experienced several thermal stress events over the last 20 years, and some reefs are exposed to chronic inputs of land-based pollution [31,32], although sedimentation rates appear to decrease in deeper reefs farther from shore [33,34]. In contrast, the FGBNMS is an isolated, relatively pristine reef system that has suffered minimally from thermal stress and land-based pollution [35]. Differences in disturbance histories, as well as abiotic factors, between these regions can potentially reveal the extent to which barnacles vary in their contribution to bioerosion across reef communities.

**Table 1:** Summary of the bioeroding activity of barnacles.

Barnacle borer	CaCO_3_ removal rate	Substratum type/host	Habitat	Source
*Lithotrya dorsalis*	2.72–5.11 kg CaCO_3_ m^-2^ year^-1^	Rock samples	Low, intertidal zone	Dineen (1990)
*L*. *dorsalis*	3.7–11.3% of total bioerosion	*Orbicella annularis*	<15 m	MacGeachy & Stearn (1976)
*L*. *dorsalis*	0.014 kg CaCO_3_ m^-2^ year^-1^	various coral species	Fringing reef	Scoffin *et al*. (1980)
*Lithotrya* sp.	0.8 cm^3^ individual^-1^ year^-1^	---	Intertidal/ limestone shore	Trudgill (1976), as reported by Glynn (1997)
*Pyrgomatidae*	23.5% of total bioerosion	*Platygyra* sp.	Rocky intertidal	Jafari *et al*. (2016)
---	3–14% of total bioerosion	*Porites* sp.	0.5–5 m	Chen *et al*. (2013)
*Pyrgomatidae*	18.7% of total bioerosion	Experimental coral substrate	<10 m	Weinstein *et al*. (2014)

All published studies investigating *Lithotrya dorsalis* are summarized above, as well as selected studies demonstrating the range of barnacle contributions to total bioerosion within a system.

The objectives of this study were to: 1) develop and ground-truth a procedure for quantifying barnacle distributions within stony coral hosts from benthic monitoring images; and 2) characterize the density of a bioeroding barnacle, *L*. *dorsalis*, with depth and *O*. *franksi* coral cover over a large spatial scale. We hypothesize that in both reef regions, barnacle density decreases with depth and scales positively with coral cover.

## Methods

### Study locations

**Fig 1:**
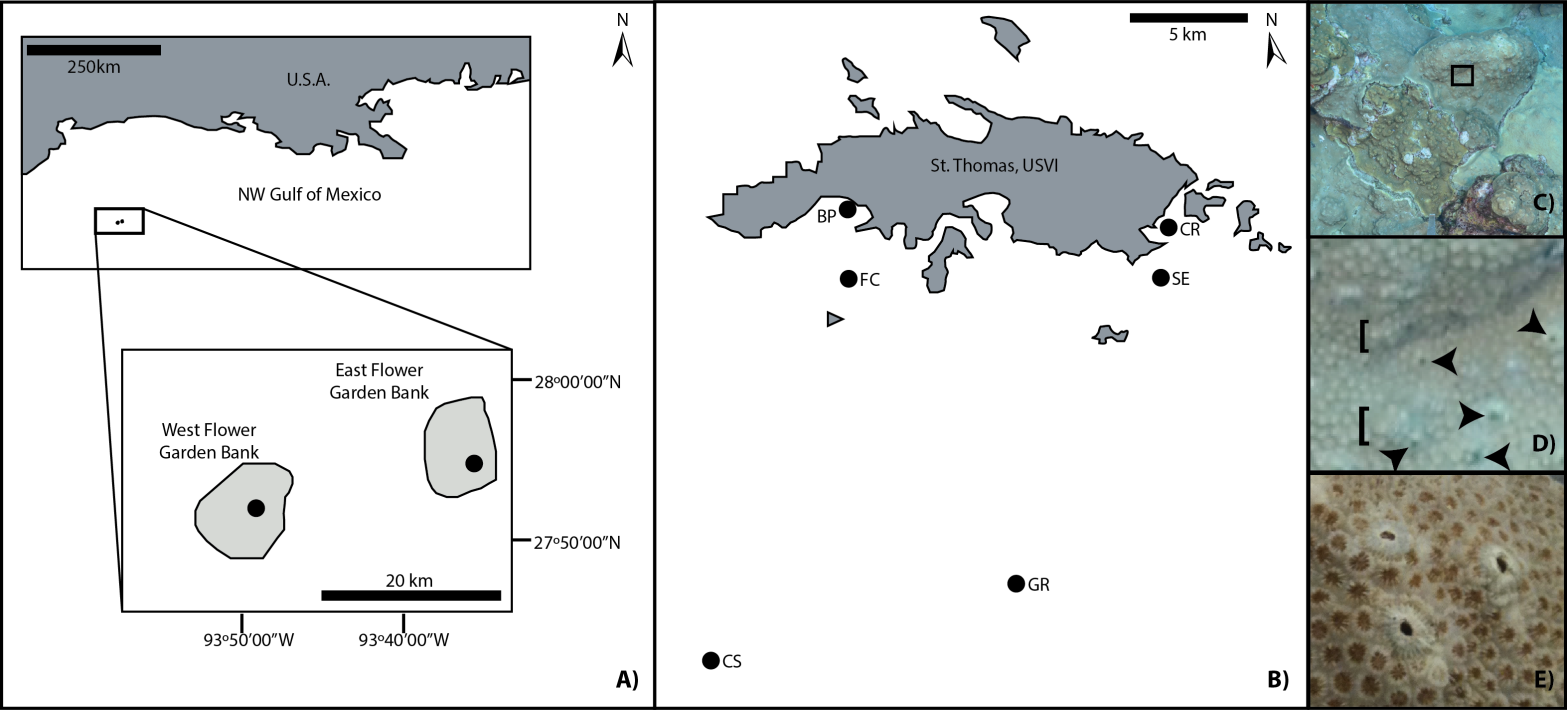
Study locations and representative benthic images. A) Geographic location of East and West Flower Garden Banks (northwest Gulf of Mexico) and general location of sampling areas (black dots); B) Geographic locations of U.S. Virgin Islands sites (BP: Black Point, CR: Coculus Rock, CS: College Shoal, FC: Flat Cay, GR: Grammanik, SE: Seahorse); C) Representative image analyzed for *Orbicella franksi* cover and barnacle (*Lithotrya dorsalis*) density; D) 200% zoom of black inset box in 1C exemplifies view used to count *L*. *dorsalis* apertures (some of which are indicated by arrowheads). These barnacle signs were readily distinguishable from colony skeletal lumps and other features (some of which are indicated by [placed to left of feature); and E) Close-up view of three *L*. *dorsalis* apertures in an *Orbicella franksi* colony from the FGB at the Houston Museum of Natural Sciences (Houston, Texas, U.S.A.).

This work was conducted in the Flower Garden Banks under National Marine Sanctuary permit #FGBNMS-2016-002 and in coordination with NOAA’s long-term monitoring program. Work in the U.S. Virgin Islands was conducted under the authority of the U.S.V.I. Department of Planning and Natural Resources, a partner in the U.S.V.I. Territorial Coral Reef Monitoring Program. This study utilized image analysis techniques to quantify the distribution and abundance of a macroboring barnacle, *L*. *dorsalis*, on colonies of a dominant reef-building coral, *O*. *franksi*. Only live *O*. *franksi* colonies were included in this analysis because this coral species is a preferred host for the barnacle and dead coral cover is rare in the FGBNMS. Barnacle attributes were compared for host corals from shallow (<30 m) to mesophotic (≥30 m) depths in 2014 from two reef systems: the East and West Flower Garden Banks (FGB), and St. Thomas, in the USVI (Fig 1A and 1B).

The FGB is located approximately 185 km south of the Texas/Louisiana border in the northwest Gulf of Mexico and constitutes the northernmost coral reef in the continental United States [36,37]. The 91 FGB monitoring locations included in our sampling were distributed across the two banks (38 EB and 53 WB sites, Fig 1A), ranged from 20 to 38 m in depth, and were no more than 21 km apart. The USVI is located in the northeastern Antilles Island arc and is bounded by the tropical western Atlantic to the north and the Caribbean Sea to the south [38]. The six sampled sites on St. Thomas, USVI (Fig 1B) ranged from 7 to 38 m in depth and were no more than 28 km apart. Both systems are dominated by *Orbicella* spp., which constitute ~25% of stony coral cover in the USVI [39] and ~30% in the FGB [35], respectively. Yet, the FGB, which is at around 45–74% coral cover, has retained approximately 10 times more stony coral than other Caribbean reefs on average [35,40,41]; this likely stems from the depth of FGB reefs, their position offshore, and their healthy grazer populations [41]. Coral cover in the USVI, which ranged from ~5 to 40% in 2014, has been depressed over time due to repeated thermal stress and corresponding bleaching events [38].

### Image analysis

In 2014 in the FGB, 4.515 m^2^ photo stations that are part of NOAA’s long term monitoring (LTM) program were photographed using a Nikon D7000 SLR camera with 16 mm lens in Sea&Sea housing with small dome port and two Inon Z240 strobes. The camera was mounted in the center of a T-shaped camera frame to maintain a distance of 2 m from the substrate [35]. The same year in the USVI, 0.389 m^2^ quadrats were photographed along three randomly selected transects out of six 10 m established transects at each monitoring site as part of the Virgin Islands Territorial Coral Reef Monitoring Program (TCRMP) [32]. Quadrats were photographed every 0.5 m for the first 5 m on the left side of each selected transect (N = 10 images per transect, 30 images per site). In the USVI, photographs were taken using a Canon G12 10.0-megapixel digital camera in an Ikelite underwater housing.

All images were processed using standardized techniques that ensured repeatability (area calculations: dx.doi.org/10.17504/protocols.io.pmvdk66; barnacle counts: dx.doi.org/10.17504/protocols.io.pmxdk7n). Briefly, *O*. *franksi* colonies from each image were identified and outlined, and total visible surface area per image was calculated using Coral Point Count with Excel extensions 4.1 Software at a zoom of 3.5x [42]. *O*. *franksi* coral cover per site was calculated by dividing the total *O*. *franksi* surface area across all images from a site by the product of the location-specific image area and total number of images per site (Table 2).

**Table 2:** Summary of images analyzed for barnacle (*Lithotrya dorsalis*) density in the coral, *Orbicella franksi*, by location and site.

	Depth (m)	Images	Images with OFR	Images with barnacles	OFR surface area (m^2^)	% OFR cover		Barnacle count
**USVI**								
Black Point	13	30	7	5	0.08	0.68	8	
Coculus Rock	7	30	3	2	0.01	0.09	3	
College Shoal	32	30	21	6	1.24	10.6	14	
Flat Cay	15	30	6	2	0.11	0.94	3	
Grammanik	38	30	4	0	0.10	0.86	0	
Seahorse	21	30	4	2	0.03	0.26	3	
**Total**	**N/A**	**180**	**45**	**17**	**1.57**	**2.24**	**31**	
**FGB**								
EB	20–24	27	27	27	41.86	34.3	14,569	
WB	20–24	41	41	41	53.25	28.8	18,595	
EB	29–38	11	10	10	12.66	25.5	2,327	
WB	29–38	12	11	11	12.62	23.3	3,450	
**Total**	**N/A**	**91**	**89**	**89**	**120.39**	**29.3**	**38,941**	

EB = East Bank; WB = West Bank; FGB = Flower Garden Banks; USVI = United States Virgin Islands; OFR = *Orbicella franksi*.

Barnacles were counted on all living *O*. *franksi* surface area within an image using the Cell Counter plugin for ImageJ (https://imagej.nih.gov/ij/plugins/cell-counter.html) at a zoom of 200%. Most barnacle burrows were identified as a small volcano-like mound culminating in a darkly shaded hole (Fig 1D and 1E). Initial *O*. *franksi* identification and all barnacle counts were performed on the Chevron DAVinCI Visualization wall (http://viz.blogs.rice.edu/) at Rice University to maximize resolution. The wall consists of stackable projection displays measuring 4 meters by 2 meters with a resolution of 7680x4320.

### *In situ* ground-truthing of barnacles

To account for potential error associated with reducing 3-D coral surfaces to 2-D photographs, from September 9–11, 2016 we collected data on *L*. *dorsalis* abundance in individual *O*. *franksi* colonies via *in situ* visual counts using a 1 m^2^ quadrat and then photographed these same colonies and calculated *L*. *dorsalis* abundance using the image analysis methods presented here. The same standardized camera set-up used in acquiring FGB LTM photographs [40] was operated by NOAA staff to photograph each colony for our methods comparison. A Bland-Altman plot was generated to explore the degree of agreement between the log-transformed barnacle counts from photos and *in situ* dives.

### Statistical analysis

A negative binomial regression was used to produce a full and a reductive (S1 Text, S2 Table, S3 Table) model of the distribution of a macroboring barnacle species with depth and coral cover across two locations. This regression was chosen because of evidence of overdispersion in the barnacle counts (i.e., variance larger than conditional mean). An offset by surface area (log(*O*. *franksi* area in m^2^)) was included in the model to reflect rate data or density as barnacle count per unit area. The continuous variables depth and *O*. *franksi* coral cover along with the categorical variable location (FGB versus USVI) and their two-way and three-way interactions were included in the full model. Coral cover per image was calculated by dividing the total *O*. *franksi* surface area in a given image by the location-specific image area (4.515 m^2^ for FGB, 0.389 m^2^ for USVI, S1 File). Three shallow FGB images did not have a reported depth and were thus assigned the average depth for shallow FGB sites (S1 File). Results were presented in terms of incident rate ratios (IRR) or the percentage increase or decrease (IRR above or below 1) in the dependent variable in terms of a unit increase in the predictor variable. A two-sample t-test showed that barnacle density (t = -0.04, df = 85.8, p = 0.97) and average *O*. *franksi* surface area per photo (t = 1.3, df = 72.3, p = 0.19) did not significantly differ between EB and WB. Given this, EB and WB site data were combined into one location for all subsequent analyses and FGB site was not included as a variable in regression analyses. USVI sites were also not included as a variable because they differed by depth (Table 2) which was included as a predictor in the regression. Raw data and calculations can be found in the S1 File. All analyses were performed using the statistical software RStudio 0.99.486.

## Results

### Barnacle density with depth

The 91 images analyzed from the FGB represented a total of 410.9 m^2^ of reef benthos, 29.3% (120.39 m^2^) of which was *O*. *franksi* cover (Table 2). From the USVI, 180 images were examined, which represented a total of 70.0 m^2^ of reef benthos, 2.2% (1.57 m^2^) of which was *O*. *franksi* cover (Table 2). Thus, total *O*. *franksi* cover was close to 100-fold greater in FGB than in USVI in 2014, based on our surveyed sites. Nearly all (98%, N = 89 of 91) of the FGB images had *O*. *franksi* cover, whereas only 25% (N = 45 of 180) USVI images had *O*. *franksi* cover.

**Fig 2:**
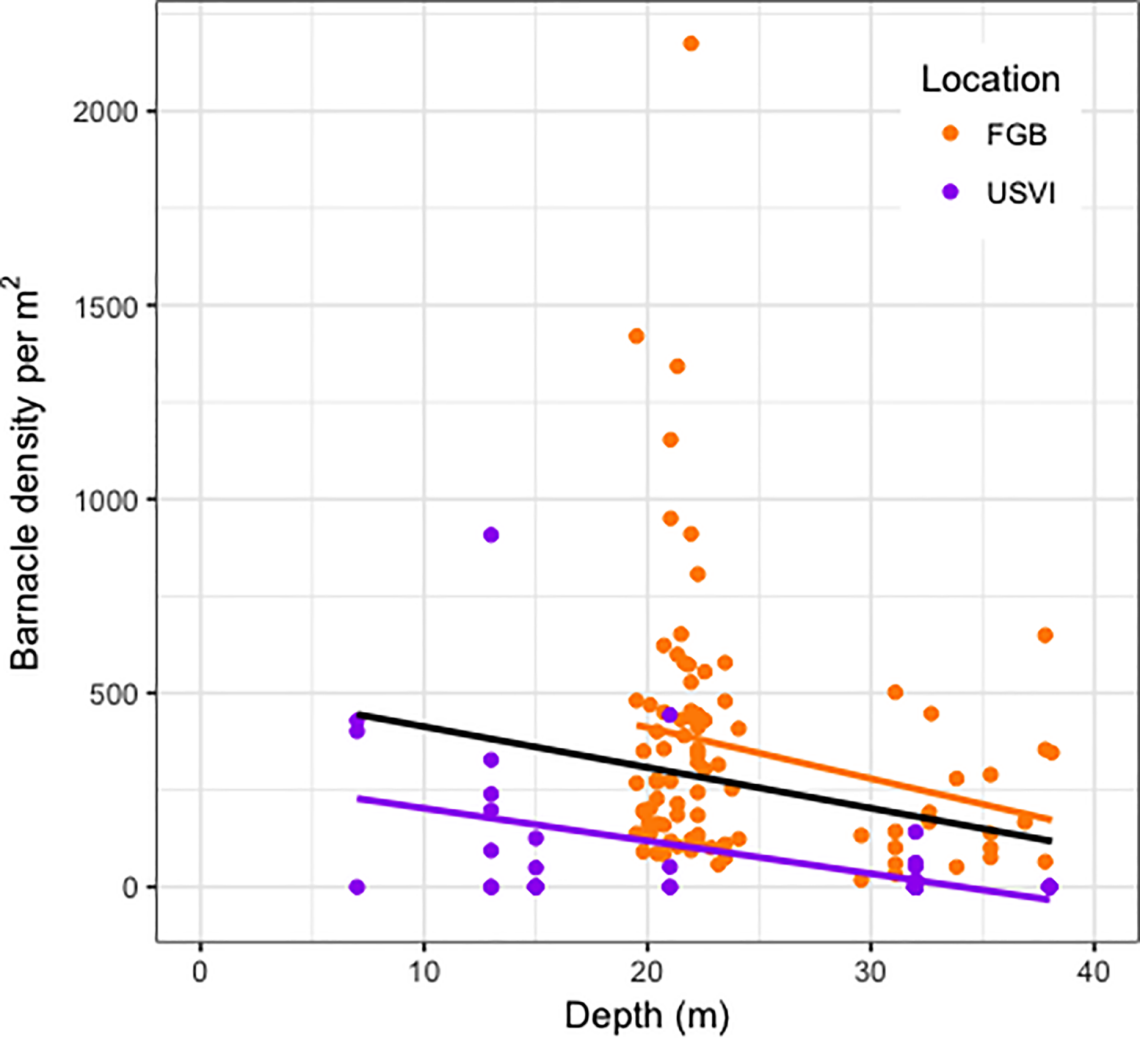
Plot of barnacle density per m^2^ decrease with depth across reef locations. Lines were added with the glm smoothing method. The black line represents the pattern for both locations combined.

A total of 38,972 *L*. *dorsalis* apertures were observed from the 135 photographs with *O*. *franksi* analyzed in this study. All 89 FGB images containing *O*. *franksi* had surface apertures (Fig 1D arrows) indicating the presence of *L*. *dorsalis*, whereas 38% (N = 17 of 45) of the USVI images containing *O*. *franksi* exhibited barnacle signs (Table 2). Average barnacle density across both locations was 260.9 per m^2^, with deep sites (100.3 per m^2^) harboring barnacle densities approximately 3.4 times lower than shallow sites (344.8 per m^2^). Negative binomial regression analysis showed that overall, a unit increase in depth corresponded to a significant 10% decrease in barnacle density (Table 3, p<0.001, Fig 2).

**Table 3:** Results of a negative binomial regression of barnacle density with depth and *Orbicella franksi* cover across locations with reported incidence rate ratios (IRR).

Variable	IRR	95% Confidence Interval
**Depth**	0.90***	**0.85, 0.96**
**OFR Coral cover**	0.94**	**0.90, 0.98**
Location–FGB	1.00	-
Location–USVI	0.38	0.05, 3.04
**Depth:OFR coral cover**	1.002*	**1.0004, 1.003**
Depth:Location–USVI	0.95	0.87, 1.03
**OFR Coral cover:Location–USVI**	0.66*	**0.45, 0.94**
**Depth:OFR Coral cover:Location–USVI**	1.01*	**1.001, 1.03**

*Orbicella franksi* area was included as an offset in the model to account for density. Depth and coral cover were included as continuous variables, whereas location was included as a factor with image as the sample unit.

**Bolded text** indicates significant main effects or interactions.

OFR = *Orbicella franksi*.

*** p < 0.001.

** p < 0.01.

* p < 0.05.

### Barnacle density with coral cover and location

Interestingly, a unit increase in *O*. *franksi* coral cover corresponded to a 6% decrease in barnacle density (Table 3, p<0.01), which suggests a negative overall relationship of barnacle density with coral cover. Barnacle distribution also exhibited trends with geographic location. For example, barnacle counts were more than 1,000-fold lower in the USVI than in the FGB (Table 2). Regression analysis of the full negative binomial model (Table 3, S1 Table) similarly showed that barnacle density in the USVI was 0.38 times that of the FGB, however the wide 95% confidence interval surrounding 1.00 indicated that this result was not significant. In contrast, results of a reductive model selection process (S1 Text, S2 Table, S3 Table) suggested that differences in barnacle density between the USVI and FGB were significant (Location-USVI IRR 0.06, 95% confidence interval 0.03, 0.10, p < 0.001). Furthermore, the average density of barnacles per m^2^ of *O*. *franksi* surface area in the USVI was nearly 4.5 times lower (79.3 per m^2^) than the density in the FGB (352.7 per m^2^, Table 4). Similarly, the effect of barnacle density decreasing with coral cover was 34% more negative in the USVI compared to the FGB, as shown with the significant interaction term of coral cover and location (p<0.05, Table 3). Finally, by location, average density at deep sites were approximately 2 (FGB) to 14 (USVI) times lower than that observed at shallow sites (Table 4). However, the two-way interaction of depth and location was not significant in the full model (Table 3) and was not included after reductive model selection (S2 Table).

**Table 4:** Summary of the distribution of a barnacle macroborer (*Lithotrya dorsalis*) and its stony coral host (*Orbicella franksi*, OFR) in the Flower Garden Banks (FGB, northwest Gulf of Mexico) and the U.S. Virgin Islands (USVI).

	FGB						USVI			
	Average	Max		Min		Median	Average	Max	Min	Median
**Shallow**										
Depth (m)	21.5 ± 1.3	24.1		19.5		21.6	14.3 ± 0.6	21	7	14
OFR surface area (m^2^)	1.4 ± 0.08	3.0		0.08		1.5	0.01 ± 0.002	0.05	0.002	0.005
% OFR cover	31.0 ± 1.7	67.4		1.9		33.2	0.33 ± 0.04	1.2	0.1	0.2
Barnacle density (number m^-2^)	398.2 ± 38.1	2174.6		57.9		318.1	163.3 ± 30.6	907.7	0	50.6
**Deep**										
Depth (m)	33.8 ± 0.3	38.1		29.6		33.8	33.0 ± 0.3	38	32	32
OFR surface area (m^2^)	1.2 ± 0.07	2.3		0.1		1.4	0.05 ± 0.007	0.2	0.001	0.04
% OFR Cover	26.7 ± 1.6	50.8		2.7		29.9	1.4 ± 0.2	6.0	0.04	1.0
Barnacle Density (number m^-2^)	205.4 ± 18.1	649.2		18.0		143.2	12.0 ± 4.0	141.9	0	0
**All Depths**										
Depth (m)	24.0 ± 4.8	38.1		19.5		22.3	24.7 ± 1.3	38	7	32
OFR surface area (m^2^)	1.4 ± 0.08	3.0		0.08		1.4	0.03 ± 0.006	0.2	0.001	0.02
% OFR Cover	30.0 ± 1.7	67.4		1.9		31.2	0.9 ± 0.1	6.0	0.04	0.6
Barnacle Density (number m^-2^)	352.7 ± 35.5	2174.6		18.0		272.5	79.3 ± 22.6	907.7	0	0

All visible *O*. *franksi* surface area per photo was included for coral cover and barnacle density calculations. OFR = *Orbicella franksi*.

The significant IRRs of ~1.00 for the two-way interaction between depth and *O*. *franksi* coral cover (p<0.05) and the three-way interactions between depth, coral cover, and location (p<0.05) imply that independent variable effects were nearly unchanged (increase by 0.2% and 1%, respectively, Table 3); significant results for these interactions were likely a product of the narrow confidence interval. The negative binomial regression had a dispersion parameter of 1.81 and coefficients are listed in S1 Table.

### Ground-truthing of image analysis method

**Fig 3:**
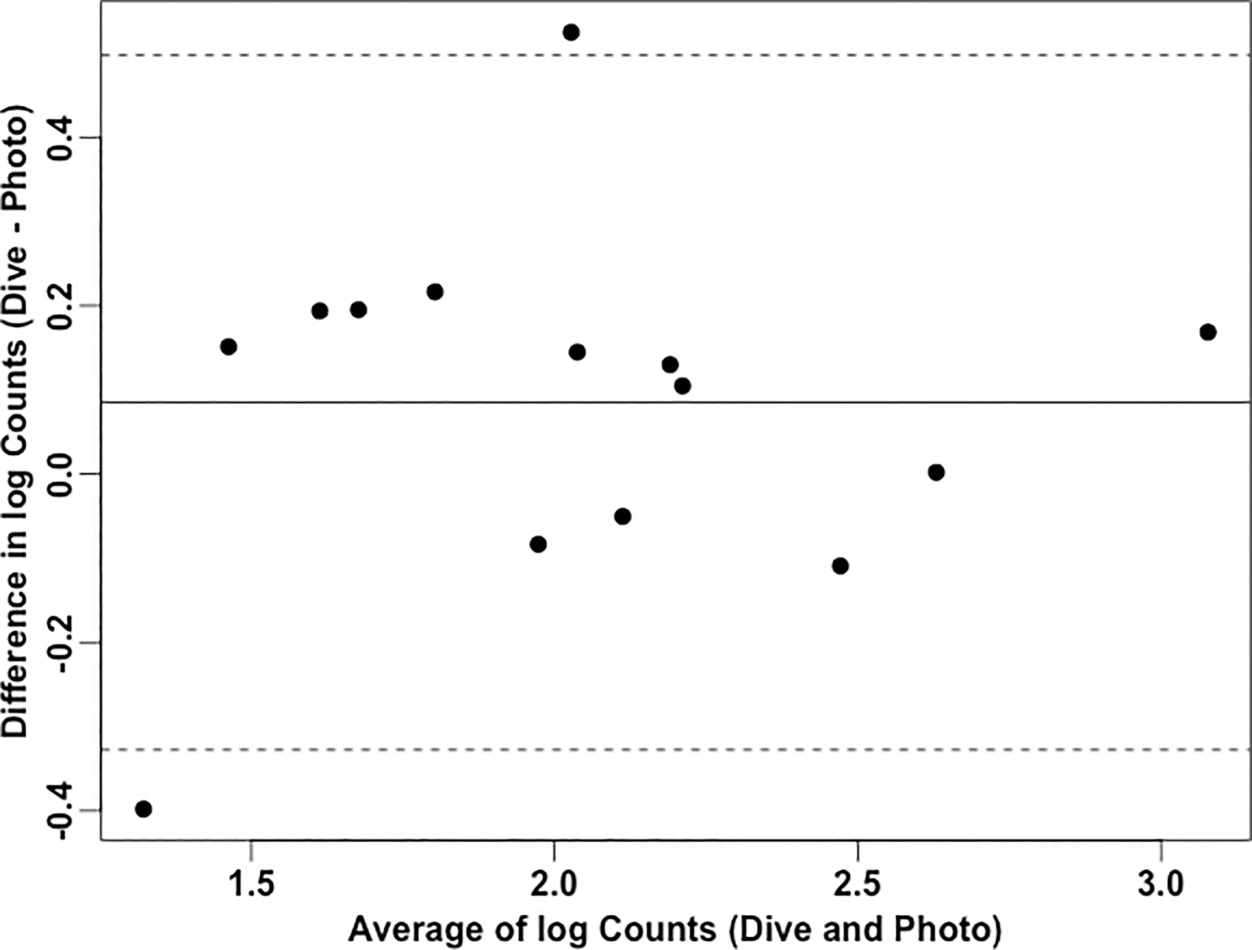
Bland-Altman plot representing the difference between log-transformed *in situ* visual census of barnacle signs from *Orbicella franksi* colonies and counts based on image analysis of the same individual corals versus the mean of these counts. The solid line represents mean differences between the two log counts of barnacle abundance; dotted lines are the upper and lower borders of the 95% limit of agreement (mean difference ± 1.96 multiplied by standard deviation of the mean difference).

To determine the accuracy of our method compared to traditional reef survey methods, we compared *in situ* visual counts of barnacle signs from individual *O*. *franksi* colonies with results from our image analysis pipeline for the same colonies. Paired data from a total of 14 FGB *O*. *franksi* colonies were collected for this process (S4 Table) and represented a total of 4.2 m^2^ of coral cover. All 14 *O*. *franksi* colonies had surface apertures indicating the presence of *L*. *dorsalis*. A total of 3,201 *L*. *dorsalis* signs were observed from these colonies based on visual census, whereas a total of 2,550 signs were identified via image analysis. The Bland-Altman plot for barnacle counts determined by the photo and *in situ* dive methods shows that *in situ* dive counts were between 0.5 to 3.1-fold the image counts for 95% of cases (Fig 3). The mean difference line is above the 0-line indicating that the image analysis method is producing barnacle counts that are more conservative than the *in situ* visual census method (Fig 3).

## Discussion

### Borer density with depth

This study demonstrates that barnacle density decreases with depth from shallow (<30m) to mesophotic (≥30 m) areas in the FGB and the USVI. Barnacle densities at shallow depths were nearly 2 to 14 times higher than in deeper areas (Table 4). Few studies investigate bioerosion by macroboring barnacles (Table 1), and even fewer quantify barnacle distributions at mesophotic depths [19,23]. MacGeachy and Stearn [15] investigated the boring activity of sponges, bivalves, worms and barnacles from a depth range of 1 to 37 m, however, and barnacles presumed to be *L*. *dorsalis* were only found on the fringing reef (<12 m) in colonies of *Montastraea (Orbicella) annularis* (Table 1). Both total bioerosion and barnacle bioerosion showed no relationship with depth, although the percent of colonies bored increased significantly with depth, most likely due to the presence of older colonies at depth compared to colonies on the fringing reef [15]. However, Moore and Shedd [16] reported a decrease in sponge boring from shallow to mesophotic depths up to 40 m, and Weinstein *et al*. [23] found that among reefs south of St. Thomas island in the USVI, total sponge, barnacle, worm, and bivalve bioerosion calculated from experimental substrate units decreased over a depth range of 9 to 45 m. Our findings also agree with studies from shallow reefs (<30 m) indicating that urchins [3,22], sponges [16,20], and polychaetes [21] decrease with depth. In contrast, other studies showed no relationship between depth and borer activity [15,24,43,44]. Macdonald and Perry [17] reported a positive trend between water depth and bioerosion and attributed the high degree of infestation across all depth zones to heavy nutrient inputs at the study site, since bioerosion has been shown to be proportional to primary productivity [28]. Goreau and Hartman [14] also reported a positive trend between water depth and sponge density but acknowledged that in their study high excavation rates could not be clearly differentiated from low rates of calcification. Whereas previous works have qualitatively characterized depth-related patterns in bioerosion [18], our study provides the first quantitative *in situ* evidence for a decrease in barnacle borer density at mesophotic depths. The depth-related patterns found in this study indicate that coral species on deeper reefs may experience reduced bioerosion relative to shallow water conspecifics. It is unclear whether this would increase net carbonate budgets on deeper reefs, however, because mesophotic corals can also experience slower skeletal accretion [45].

Various abiotic and biotic covariates may be driving or contributing to decreasing barnacle density with depth. For instance, temperature, pH, and primary productivity co-vary with depth, and water column height is strongly associated with light, waves, and turbulence [46,47]. In fact, various studies report a negative relationship between bioerosion and pH [48,49], and bioerosion was found to be higher in corals with low aragonite saturation states [47]. Bioerosion rates can also be associated with various biological drivers like benthic cover and herbivore biomass since borers can have complex settlement interactions with algae and secondary calcifiers [50].

### Differences in barnacle density with coral cover and reef region

Across the two reef regions, barnacle density scaled negatively with increasing coral cover (Table 3). This non-linear relationship suggests that barnacle populations are not strictly space-limited in their distribution and settlement opportunities. In fact, borer colonization and density may depend on complex relationships with variables such as colony morphology, current, and sedimentation [24]. This negative relationship was strongest in the USVI, suggesting that barnacles are less successful at colonizing *O*. *franksi* in this region and are not keeping up with available coral substrate to the same degree as barnacles in the FGB. Additional studies exploring the spatial distribution of macroborers across individual colonies are needed. For example, distance to nearest neighbor measurements [25,51] can be used to characterize overdispersion, clustering, preferential settlement or other patterns in macroborer colonization of host substrate. These types of analyses can be performed in conjunction with the image analysis approach described here to provide a better understanding of the population ecology of macroboring barnacles.

It is unclear whether reef location alone is a biologically meaningful factor in predicting barnacle density, since the full and reductive models presented here provide conflicting statistical results. The USVI harbored a relatively low abundance of *L*. *dorsalis* in *O*. *franksi*, which may reflect the lower available coral cover for barnacle settlement at this location. In contrast, the large number of barnacles observed in the FGB strongly demonstrates that this region experiences high levels of barnacle bioerosion on at least one of its dominant reef-building coral species. Risk *et al*. [52] suggest that intense bioerosion may be an indicator of stress in corals, since colonies least able to protect themselves from borer settlement and growth might exhibit the highest rates of bioerosion. Others have proposed that high rates of macrobioerosion may serve as a bioindicator of decreasing water quality [53–55]. These two hypotheses are not necessarily mutually exclusive. However, the disturbance history of the FGB does not suggest that corals in the region experienced stress in recent years up to and including 2014. The FGB generally experiences high coral cover, low thermal stress, and low nutrient pollution [36]. NOAA LTM water quality data from the FGB measures for ammonia, chlorophyll-a, nitrate, nitrite, phosphorous, and nitrogen [56], and anecdotal data from monitoring in 2014 showed that all parameters were below detectable levels excluding nitrate, which averaged 0.06 mg L^-1^. In contrast, many reefs in the USVI are reported to experience nearshore nutrient pollution with high chlorophyll concentrations and turbidity [32]. The temperature regimes of the two regions also differ greatly. In the FGB, the coolest temperatures (~20°C) were observed at the deepest sites [35]. Although both regions have experienced temperatures up to 30°C [32,35], mesophotic reefs in the USVI more commonly experience high temperature thermal stress and the lowest reported temperatures are ~24°C [32,38]. The high abundance of barnacles in the FGB may be explained by the preference of *L*. *dorsalis* for cooler, nutrient poor waters. To fully understand the prevalence of *L*. *dorsalis* in the FGB, additional abiotic and biotic factors should be directly investigated. For instance, grazing intensity has been suggested to partially regulate macrobioeroding communities [57]. The relative isolation of the FGB may also limit the recruitment of some dominant bioeroding groups to this region [35], potentially releasing *L*. *dorsalis* from some competitive interactions in the FGB. Further investigations into spatial heterogeneity in the distribution of *L*. *dorsalis* and other borers among reef regions are needed, particularly at sites with similar depth and coral assemblages (e.g., Cuban reefs).

### Barnacle macroborers: Their influence on coral reefs

The results presented in this study must be considered in the context of total bioerosion within each system. In most studies (Table 1), barnacle bioerosion only accounts for ~4–20% of total bioerosion. However, Jafari *et al*. [58] found that burrowing barnacles of the family *Pyrgomatidae* were the most effective eroding organisms in colonies of live *Platygyra* coral, contributing about 23.5% of total erosion of this host species; another study reported these barnacles as the exclusive borers from *Platygyra* [59]. Although it was beyond the scope of this study to calculate the percent contribution of *L*. *dorsalis* to total bioerosion, the impressive number of barnacle signs recorded from the FGB (Table 2), and the fact that borer diversity is typically reduced on deeper reefs [59], suggests that *L*. *dorsalis* may represent a significant component of total bioerosion in *O*. *franksi* colonies, and perhaps on FGB reefs. In contrast, in the USVI, barnacles are most likely a minor contributor to total bioerosion (Table 2). Weinstein *et al*. [23] reported that bioerosional grazing dominated substrate modification in USVI reefs down to 30 m, whereas sponges were the dominant borers in reefs between 35 and 50 m. In fact, many studies have reported that sponges were the main contributors to bioerosion on deeper reef-fronts (15–50 m) [14,19]. For a more complete picture of total bioerosion within the two systems, our procedure could be applied to include multiple macrobioeroding groups such as worms and bivalves (e.g., *Spirobranchus giganteus*, *Lithophaga* sp.) and to multiple host coral species.

### Image analysis approaches for characterizing macroborers

Reef managers may find utility in tracking borer abundances over space and time since these organisms can be used as indicators of disturbance [1]. The image analysis approach presented here can be widely applied to characterize macroborers that create distinctive visual signs on coral colonies, including bivalves, barnacles, and polychaetes. It should be noted that the barnacle counts and densities presented in this study may be overestimates as these image analysis methods do not differentiate between living and dead barnacles within a coral host. However, this potential source of error is also an issue when conducting *in situ* visual surveys of macrobioeroding barnacles and other traditional field methods. Since this issue applies equally to all observations in this study, it does not impact the detection of patterns in barnacle distribution with depth, coral cover or reef region.

Image analysis of *in situ* coral colonies complements colonization and bioerosion rate data from traditional field methods such as deployment of experimental carbonate blocks, sample casts or molds, and coring of live or dead coral [8,13,60]. These latter methods, however, can be labor-intensive and require minimum deployments of two to five years before differences in bioerosion intensity between sites are observed [23,43,57]. In combination with the image analysis method of barnacle density presented here, measurements of burrow volume per *L*. *dorsalis* individual could generate a reference rate of *L*. *dorsalis* erosion of calcium carbonate substrate for the FGB, USVI and other locations.

Our image analysis pipeline can additionally be applied to longitudinal photo datasets to calculate macroborer densities in the past and present and to develop models to predict borer impacts in the future. In the FGB, the current standardization of long-term monitoring methods and equipment [56] provides an ideal opportunity to leverage image analysis approaches to characterize the distributions of some borers (e.g., barnacles, Christmas tree worms) and their impacts on the system over time. For instance, over a long timescale (>10 years), our procedure can be applied to study how bioerosion affects the growth and trajectories of individual coral colonies or different host species. Application to a long time-series could also reveal patterns in macroborer recruitment and settlement over time. The methods described here can also be applied to collections of photos from existing Long Term Monitoring (LTM) programs on other coral reefs. Reef managers may find utility in tracking borer abundances over space and time since these organisms can be used as indicators of disturbance and changes in water column productivity [1,28]. With the computer software used in this study, identification of barnacle macroborers is reliable (Fig 3), fast, and easy. *In situ* ground-truthing demonstrated that the image analysis pipeline produces a conservative estimate of barnacle abundance as compared to visual counts on scuba using a quadrat.

## Conclusions

This study demonstrates that barnacle density decreases with increasing depth and *O*. *franksi* cover on two reefs that vary in terms of their disturbance regimes and some biotic factors. The FGB is minimally disturbed compared to St. Thomas (USVI) reefs, has a higher *O*. *franksi* percent cover and, interestingly, a high abundance of a barnacle borer. This quantitative study adds to the limited data available on macroborer distribution and density on deep reefs. It also provides some of the first evidence that boring barnacles may be major players in the carbonate budget of some coral species, and possibly, reefs. Additional research is needed to determine whether macrobioeroding barnacles are highly abundant in any other comparable reef assemblages in the Gulf of Mexico and Caribbean, and to understand the factors that drive spatial heterogeneity in the distribution of macroborers.

## Supporting information

S1 File Metadata and results for individual images analyzed in this study. (CSV)

S1 Text Description of reductive model selection method. (PDF)

S1 Table Results of negative binomial regression model on barnacle count data with reported coefficients. Model included all variables and interactions with an offset by *Orbicella franksi* area per photo. Results include standard errors, test statistics and p-values. **Bolded text** indicates significant main effects or interactions.
(PDF)

S2 Table Results of a negative binomial regression model with reductive model selection of barnacle density with depth and *Orbicella franksi* cover across locations with reported incidence rate ratios (IRR). *Orbicella franksi* area was included as an offset in the model to account for density. Depth and coral cover were included as continuous variables, whereas location was included as a factor with image as the sample unit. Main effects and interactions were evaluated using a log likelihood test to determine whether they impacted model results, and thus warranted inclusion in the final iteration of the model. **Bolded text** indicates significant main effects or interactions. OFR = *Orbicella franksi*. *** p < 0.001; ** p < 0.01; * p < 0.05.
(PDF)

S3 Table Results of negative binomial regression model with reductive model selection of barnacle density with depth and *Orbicella franksi* cover across locations with reported coefficients. *Orbicella franksi* area was included as an offset in the model to account for density. Depth and coral cover were included as continuous variables, whereas location was included as a factor with image as the sample unit. Main effects and interactions were evaluated using a log likelihood test to determine whether they impacted model results, and thus warranted inclusion in the final iteration of the model. **Bolded text** indicates significant main effects or interactions. OFR = *Orbicella franksi*.
(PDF)

S4 Table Results of *in situ* ground-truthing of image analysis pipeline. Counts were made of the barnacle *Lithotrya dorsalis* in colonies of *Orbicella franksi* using a 1 m^2^ quadrat.
(PDF)
