# Supplementary material for: Depth and coral cover drive the distribution of a coral macroborer across two reef systems
Source: PLoS One. 2018 Jun 20;13(6):e0199462. doi: 10.1371/journal.pone.0199462 (PMC6010239; doi:10.1371/journal.pone.0199462)
Supplement: S1 Text — (PDF) [file pone.0199462.s002.pdf]

Model selection was performed using a reductive, leave-one-out method. First, all main effects were evaluated with a log likelihood test to determine whether they impacted model results, and thus warranted inclusion in the final iteration of the model. Next, all main effects and two-way interactions were similarly evaluated. The three-way interaction was not evaluated for the reductive model selection since several two-way interactions were eliminated from the model after log likelihood comparison.
