# Supplementary material for: Depth and coral cover drive the distribution of a coral macroborer across two reef systems
Source: PLoS One. 2018 Jun 20;13(6):e0199462. doi: 10.1371/journal.pone.0199462 (PMC6010239; doi:10.1371/journal.pone.0199462)
Supplement: S1 Table — Model included all variables and interactions with an offset by Orbicella franksi area per photo. Results include standard errors, test statistics and p-values. Bolded text indicates significant main effects or interactions. (PDF) [file pone.0199462.s003.pdf]

	Estimate	Std. Error	z value	Pr(>|z|)
**(Intercept)**	**8.58**	**0.74**	**11.45**	**< 0.001**
**Depth**	**-0.10**	**0.03**	**-3.47**	**< 0.001**
**OFR coral cover**	**-0.06**	**0.02**	**-2.61**	**< 0.01**
Location – USVI	-0.98	1.04	-0.94	p=0.35
**Depth:OFR coral cover**	**0.002**	**0.0009**	**2.30**	**< 0.05**
Depth:Location – USVI	-0.05	0.04	-1.22	p=0.22
**OFR coral cover: Location – USVI**	**-0.41**	**0.16**	**-2.51**	**<0.05**
**Depth:OFR coral cover:Location – USVI**	**0.01**	**0.005**	**2.37**	**<0.05**
