# Supplementary material for: Depth and coral cover drive the distribution of a coral macroborer across two reef systems
Source: PLoS One. 2018 Jun 20;13(6):e0199462. doi: 10.1371/journal.pone.0199462 (PMC6010239; doi:10.1371/journal.pone.0199462)
Supplement: S2 Table — Orbicella franksi area was included as an offset in the model to account for density. Depth and coral cover were included as continuous variables, whereas location was included as a factor with image as the sample unit. Main effects and interactions were evaluated using a log likelihood test to determine whether they impacted model results, and thus warranted inclusion in the final iteration of the model. Bolded text indicates significant main effects or interactions. OFR = Orbicella franksi. *** p < 0.001; ** p < 0.01; * p < 0.05. (PDF) [file pone.0199462.s004.pdf]

Variable	IRR	95% Confidence Interval
**Depth**	**0.89*** **	**0.85, 0.92**
**OFR Coral cover**	**0.93*** **	**0.90, 0.96**
Location – FGB	1.00	-
**Location – USVI**	**0.06*** **	**0.03, 0.10**
**Depth:OFR Coral cover**	**1.003*** *	**1.001, 1.004**
