# Supplementary material for: Depth and coral cover drive the distribution of a coral macroborer across two reef systems
Source: PLoS One. 2018 Jun 20;13(6):e0199462. doi: 10.1371/journal.pone.0199462 (PMC6010239; doi:10.1371/journal.pone.0199462)
Supplement: S3 Table — Orbicella franksi area was included as an offset in the model to account for density. Depth and coral cover were included as continuous variables, whereas location was included as a factor with image as the sample unit. Main effects and interactions were evaluated using a log likelihood test to determine whether they impacted model results, and thus warranted inclusion in the final iteration of the model. Bolded text indicates significant main effects or interactions. OFR = Orbicella franksi. (PDF) [file pone.0199462.s005.pdf]

	Estimate	Std. Error	z value	Pr(>|z|)
**(Intercept)**	**9.08**	**0.54**	**16.71**	**< 0.001**
**Depth**	**-0.12**	**0.02**	**-5.60**	**< 0.001**
**OFR Coral cover**	**-0.07**	**0.02**	**-3.76**	**< 0.001**
**Location – USVI**	**-2.84**	**0.26**	**-10.95**	**< 0.001**
**Depth: OFR Coral cover**	**0.002**	**0.0008**	**3.27**	**< 0.01**
