# Supplementary material for: Depth and coral cover drive the distribution of a coral macroborer across two reef systems
Source: PLoS One. 2018 Jun 20;13(6):e0199462. doi: 10.1371/journal.pone.0199462 (PMC6010239; doi:10.1371/journal.pone.0199462)
Supplement: S4 Table — Counts were made of the barnacle Lithotrya dorsalis in colonies of Orbicella franksi using a 1 m2 quadrat. (PDF) [file pone.0199462.s006.pdf]

Colony	Site	Photo count	*In situ* count	Difference
1	WB	137	122	15
2	WB	143	182	-39
3	WB	966	1424	-458
4	WB	49	164	-115
5	EB	103	85	18
6	EB	30	12	18
7	EB	37	58	-21
8	EB	24	34	-10
9	EB	32	50	-18
10	EB	91	127	-36
11	EB	425	427	-2
12	EB	333	259	74
13	EB	48	79	-31
14	EB	132	178	-46
**Total**		**2550**	**3201**	**-651**
